# Supplementary material for: Eating Disorders in an Immigrant Population: Are Clinical Features and Treatment Outcomes Different from the Native-Born Spanish Population?
Source: Nutrients. 2025 Dec 14;17(24):3914. doi: 10.3390/nu17243914 (PMC12735791; doi:10.3390/nu17243914)
Supplement: Supplementary file 1 [file nutrients-17-03914-s001.zip › nutrients-4003601-supplementary.pdf]

Supplementary Materials

Table S1. Origin of patients in the immigrant group

**Origin**	***n***	**%**
Latin America	112	71.34%
Rest of Europe	21	13.38%
Africa	13	8.28%
Eastern Europe	10	6.37%
Asia	1	0.64%
